# Differential G protein subunit expression by prostate cancer cells and their interaction with CXCR5

**DOI:** 10.1186/1476-4598-12-64

**Published:** 2013-06-18

**Authors:** Christelle P El-Haibi, Praveen Sharma, Rajesh Singh, Pranav Gupta, Dennis D Taub, Shailesh Singh, James W Lillard, Jr

**Affiliations:** 1 Department of Pathology, Beth Israel Deaconess Medical Center, Harvard Medical School, Boston, MA, USA; 2 School of Natural Sciences, Center of Life Sciences, Central University of Jharkhand, Brombe, Ranchi, India; 3 Department of Microbiology, Biochemistry, & Immunology, Morehouse School of Medicine, Atlanta, GA, USA; 4 Laboratory of Immunology, National Institute on Aging, National Institute of Health, Bethesda, Maryland, USA

**Keywords:** Prostate cancer, Chemokine, G proteins, G protein-coupled receptor

## Abstract

**Background:**

Prostate cancer (PCa) cell lines and tissues differentially express CXCR5, which positively correlate with PCa progression, and mediate PCa cell migration and invasion following interaction with CXCL13. However, the differential expression of G protein α, β, and γ subunits by PCa cell lines and the precise combination of these proteins with CXCR5 has not been elucidated.

**Methods:**

We examined differences in G protein expression of normal prostate (RWPE-1) and PCa cell lines (LNCaP, C4-2B, and PC3) by western blot analysis. Further, we immunoprecipitated CXCR5 with different G protein subunits, and CXCR4, following CXCL13 stimulation. To investigate constitutive coupling of CXCR5 with CXCR4 and PAR-1 we performed invasion assay in PCa cells transfected with G_αq/i2_ or G_α13_ siRNA, following CXCL13 treatment. We also investigated Rac and RhoA activity by G-LISA activation assay in PCa cells following CXCL13/thrombin stimulation.

**Result:**

Of the 22 G proteins studied, G_αi1-3_, G_β1-4_, G_γ5_, G_γ7_, and G_γ10_ were expressed by both normal and PCa cell lines. G_αs_ was moderately expressed in C4-2B and PC3 cell lines, G_αq/11_ was only present in RWPE-1 and LNCaP cell lines, while G_α12_ and G_α13_ were expressed in C4-2B and PC3 cell lines. G_γ9_ was expressed only in PCa cell lines. G_α16_, G_β5_, G_γ1-4_, and G_γ13_ were not detected in any of the cell lines studied. Surprisingly, CXCR4 co-immunoprecipitated with CXCR5 in PCa cell lines irrespective of CXCL13 treatment. We also identified specific G protein isoforms coupled to CXCR5 in its resting and active states. G_αq/11/_G_β3/_G_γ9_ in LNCaP and G_αi2/_G_β3/_G_γ9_ in C4-2B and PC3 cell lines, were coupled to CXCR5 and disassociated following CXCL13 stimulation. Interestingly, G_α13_ co-immunoprecipitated with CXCR5 in CXCL13-treated, but not in untreated PCa cell lines. Inhibition of G_αq/i2_ significantly decreased the ability of cells to invade, whereas silencing G_α13_ did not affect CXCL13-dependent cell invasion. Finally, CXCL13 treatment significantly increased Rac activity in G_αq/i2_ dependent manner, but not RhoA activity, in PCa cell lines.

**Conclusions:**

These findings offer insight into molecular mechanisms of PCa progression and can help to design some therapeutic strategies involving CXCR5 and/or CXCL13 blockade and specific G protein inhibition to abrogate PCa metastasis.

## Background

G protein-coupled receptors (GPCRs) are divided into three broad classes based on the similarity of the transmembrane sequences and the nature of their ligand [1]. Chemokine receptors are categorized under the superfamily of Class A Rhodopsin-like GPCRs [2]. GPCRs interact with heterotrimeric guanine nucleotide-binding proteins (G proteins) composed of α, β, and γ subunits present on the inner surface of the plasma membrane. After ligand binding, the receptor elicits a conformational alteration resulting in the exchange of guanosine diphosphate (GDP) for guanosine triphosphate (GTP) by the G_α_ subunit. This leads to heterotrimer dissociation and stimulation of downstream effector molecules to initiate intracellular signaling cascades [1,3,4]. G_α_ subunits are divided into four families G_αs_, G_αi_, G_αq/11_, and G_α12/13_ based on sequence homology and functional similarities. G_αs_ proteins are known to stimulate adenylyl cyclases (AC), while G_αi_ proteins inhibit AC and activate phosphodiesterases. Alternatively, G_αq/11_ proteins regulate the activity of phosphatidylinositol-specific phospholipases to generate lipid second messengers, and G_α12/13_ proteins regulate the small guanine triphosphate (GTPases). On the other hand, G protein β and γ subunits function as a tightly associated complex to modulate the activity of several effectors including AC, protein tyrosine kinases (*e*.*g*., Src family tyrosine kinases), phosphoinositide-3 kinase (PI3K) γ, GPCR kinases (GRKs), and Ca^+2^ as well as K^+^ ion channels [4,5].

G_α_ subunits are encoded by 17 genes (*Gnas*, *Gnasxl*, *Gnal*, *Gnai1*, *Gnai2*, *Gnai3*, *Gnao*, *Gnaz*, *Gnag*, *Gnat1*, *Gnat2*, *Gnaq*, *Gna11*, *Gna14*, *Gna15*, *Gna12*, and *Gna13*). There are five known genes encoding G_β_ subunits (*Gnb1*, *Gnb2*, *Gnb3*, *Gnb4*, and *Gnb5*) and 12 genes encoding G_γ_ subunits (*Gngt1*, *Gngt2*, *Gng2*, *Gng3*, *Gng4*, *Gng5*, *Gng7*, *Gng8*, *Gng10*, *Gng11*, *Gng12*, and *Gng13*) [3,6,7]. A large number of potential combinations of G_α/β/γ_ heterotrimers can form; however, not all associations are functional and they vary in their affinity for distinct GPCRs [8,9]. G proteins also exhibit tissue-specific expression. Most G proteins are ubiquitously present in several tissues, but a smaller subset is confined to specialized cell types [7,10].

Several studies have reported the role of G proteins in different human diseases [11]. Comparatively, less is known regarding the expression of these signaling proteins by PCa cells. PCa cells express a repertoire of chemokine receptors that contribute to disease progression and metastasis [12,13]. In this regard, we have shown that PCa cell lines differentially express CXCR5, and this expression positively correlates with the ability of cell lines to migrate and invade extracellular matrix components following interaction with CXCL13 [14,15]. To our knowledge, neither the differential expression of G protein α, β, and γ subunits by PCa cell lines nor specific G protein interactions with CXCR5 have been described. Here, we elucidate the differences in G protein isoforms expressed by normal and tumorigenic prostate cell lines. We also identified the specific G protein isoforms coupled to CXCR5 in the presence or absence of CXCL13 stimulation.

## Results

### Endogenous expression of G_α_ protein subunits by PCa cells

**Figure 1:**
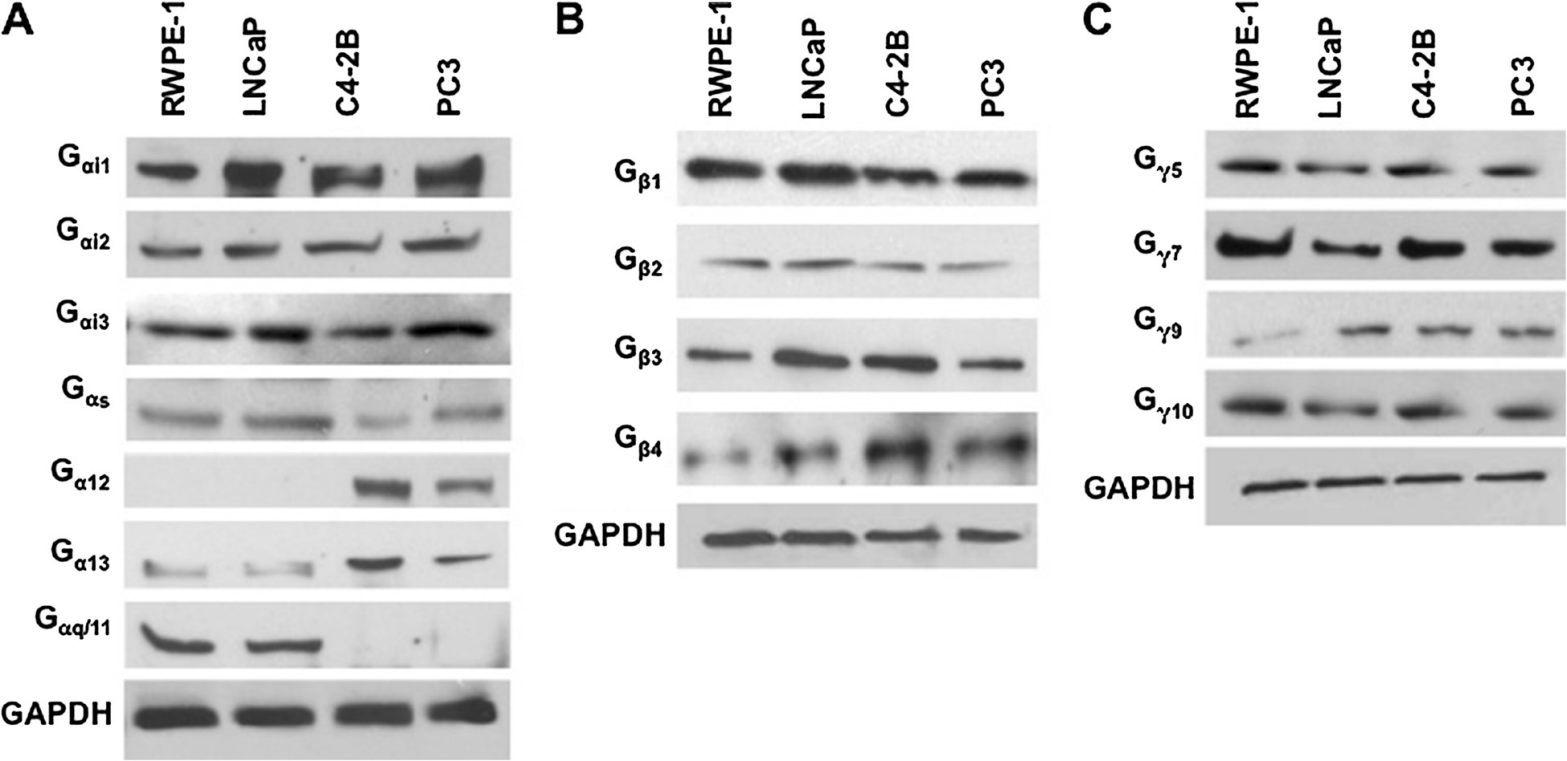
**G protein α subunit isoforms expressed by PCa cell lines.** Equal protein amounts (50 μg) from RWPE-1, LNCaP, C4-2B, and PC3 cell lysates were resolved by SDS-PAGE and the expression of (**A**) G_α_ (**B**) G_β_ and (**C**) G_γ_ subunits were determined by immunoblot. The blots in each panel were re-probed to stain different G-proteins subunits. GAPDH served as loading control. All experiments were repeated three times with unvarying results.

In light of the diversity of G protein isoforms and functions, we revealed the differential expression of G protein α, β, and γ isoforms by Western blot analysis of total lysates of untreated PCa and normal cell lines. Our results show that G_αi_ subunit (isoforms 1, 2, and 3) are widely expressed by RWPE-1, LNCaP, C4-2B, and PC3 cell lines (Figure 1A). The G_αs_ subunit was expressed by all cell lines studied, with reduced levels in C4-2B and PC3 cell lines. G_α12_ was expressed by hormone refractory cell lines C4-2B and PC3, but was absent in RWPE-1 and LNCaP cell lines. G_α13_ was unequally distributed among the four cell lines examined, showing elevated levels in C4-2B and PC3 cell lines (Figure 1A). The G_αq/11_ subunit was confined to the androgen-dependent cell lines - RWPE-1 and LNCaP and not detected in hormone refractory cell lines, C4-2B and PC3 (Figure 1A). Lastly, G_α16_ was not detected in any of the tested cell lines (data not shown), probably due to its specificity for hematopoietic cells [16].

### Endogenous expression of G_βγ_ -protein subunits by prostate cells

Except for the G_β5_ isoform (data not shown), which reported to be largely expressed by brain tissue [5,9], all other G_β_ isoforms were present in all prostate cell lines examined (Figure 1B). The expression of G_γ_ subunits exhibited a distinctive pattern where only isoforms [5,7,9,10] were detected in the cell lines studied. As expected, G_γ1-4_ and G_γ13_ were not detected in any of the cell lines tested (data not shown) (Figure 1C), because they have previously shown to be confined to retinal rods, brain tissue, and taste buds respectively [3]. Interestingly, G_γ9_ was expressed at very low levels in the normal prostate cell line, but was significantly expressed in all of the PCa cell lines tested.

### Specific G proteins coupled to CXCR5 in PCa cell lines

**Figure 2:**
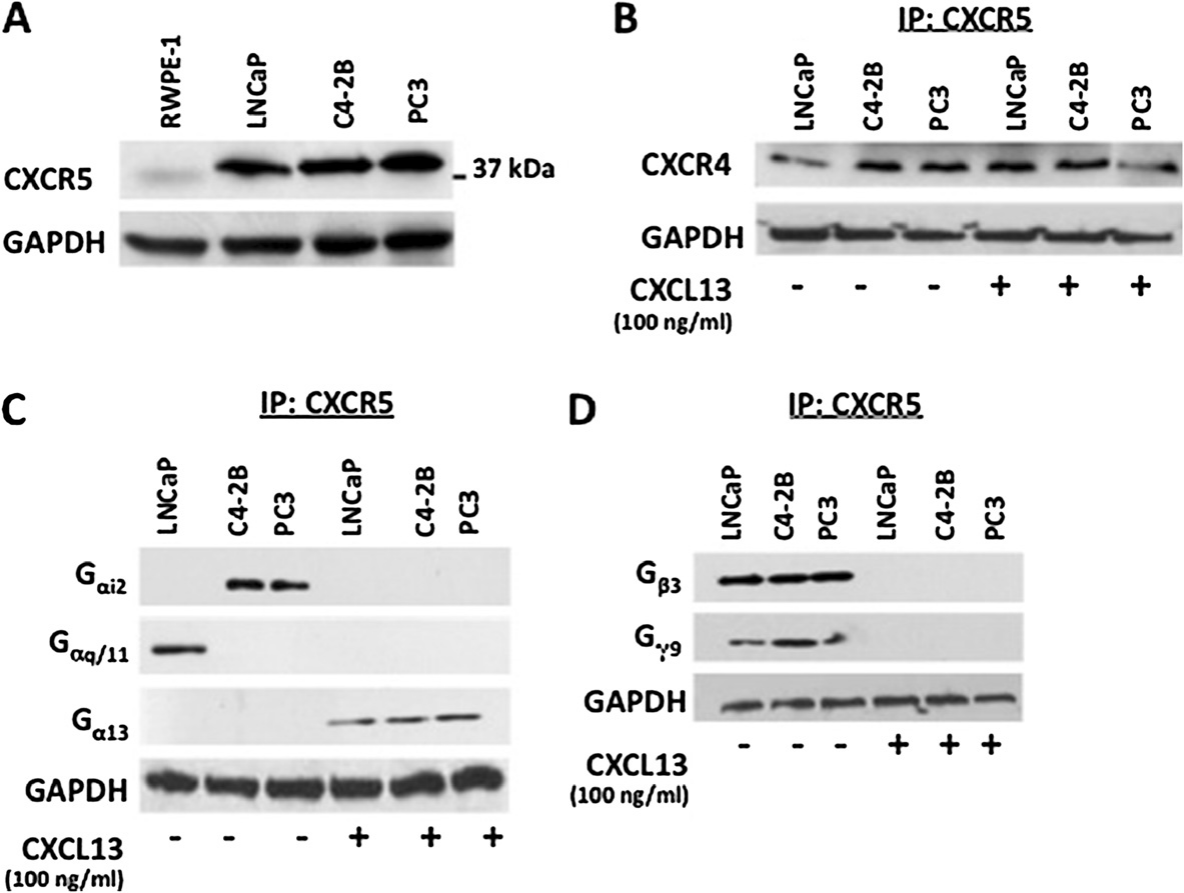
**Expression of CXCR4, CXCR5 and associated G proteins in PCa cell lines.** (**A**) Equal protein amounts (50 μg) from RWPE-1, LNCaP, C4-2B, and PC3 cell lysates were resolved by SDS-PAGE and the expression of CXCR5 (37 kDa) was determined by immunoblot. (**B**) Western blot analysis of CXCR4 expression with and without CXCL13 treatment. (**C**) & (**D**) Cell lines were treated with or without CXCL13 and lysed. CXCR5 was immunoprecipitated (IP) to pull down associated proteins from total cell lysates. The IP cell lysates were resolved by SDS-PAGE and the expression of (**C**) G_αi1_, G_αi2_, G_αi3_, G_αs_, G_αq/11_, G_α12_, G_α13_ (**D**) G_β1_, G_β2_, G_β3_, G_β4_, and G_γ5_, G_γ7_, G_γ9_, G_γ10_ were examined by immunoblot. The blots in panel C and D were stripped each time and re-probed to stain different G_α_, G_β_, and G_γ_ protein subunits. In all the experiments, GAPDH served as loading control. All experiments were repeated three times with unvarying results.

It is now well established that chemokine receptors are often up-regulated and potentially influence the tumor behavior in a variety of human cancers including prostate cancer. Here, we demonstrate that CXCR5 is highly expressed by PCa cell lines (LNCaP, C4-2B, and PC3), but in low to undetectable amount by the normal prostate cell line, RWPE-1 (Figure 2A). Chemokine receptors are usually, but not exclusively, coupled to G_αi_ subclass of G proteins [17]. In this study, we demonstrate that only G_αi2_ co-immunoprecipitated with CXCR5 in untreated C4-2B and PC3 cell lines in the absence of agonist, while G_αq/11_ associates with CXCR5 in untreated LNCaP cells. G_α13_ co-immunoprecipitated with CXCR5 in all three PCa cell lines treated with CXCL13, but was not detected in untreated cells (Figure 2C). G_β3_ and G_γ9_ co-immunoprecipitated with CXCR5 in the absence of CXCL13 in all PCa cell lines used (Figure 2D). This G_β3/γ9_ complex was not detected following CXCL13 stimulation indicating its ligand-induced dissociation from the receptor. The other G_α (i1, i3),_ G_s,_ G_α12_, G_β (1, 2, 4)_ and G_γ (5, 7, 10)_ subunits which were detected in PCa cell lines (Figure 1B and 1C) were not co-immunoprecipitated with CXCR5 in presence or absence of agonist (data not shown).

### Validation and significance of G_αq/11/_G_β3/_G_γ9_ and G_αi2/_G_β3/_G_γ9_ binding to CXCR5 in LNCaP, and C4-2B, and PC3 cell lines respectively

**Figure 3:**
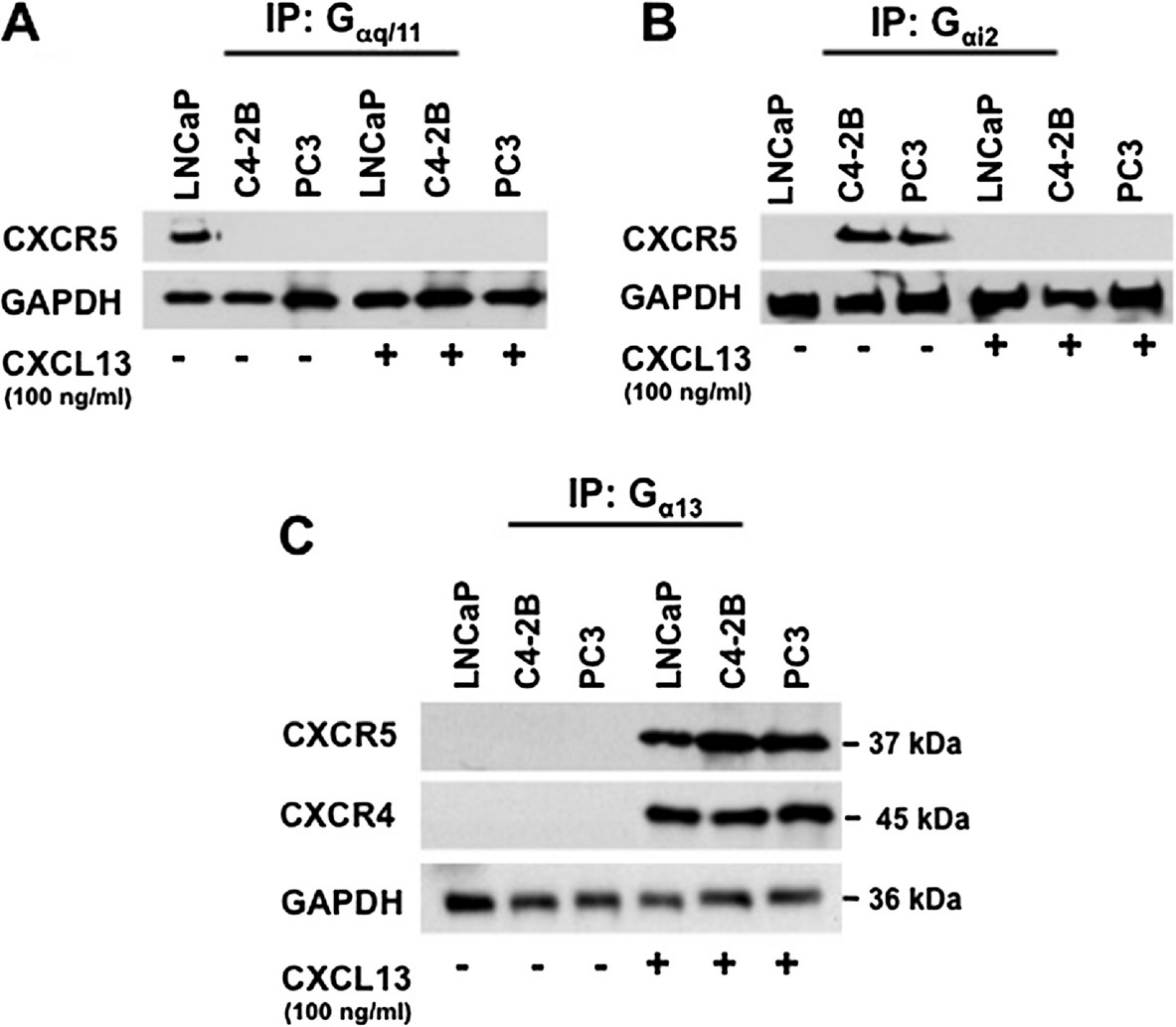
**Validation of G**_**αq/11**_**, G**_**αi2**_**, and G**_**α13 **_**protein association with CXCR5.** Cell lines were treated with or without CXCL13 and lysed. (**A**) G_αq/11_ and (**B**) G_αi2_ were immunoprecipitated (IP) from total cell lysates. The IP cell lysates were resolved by SDS-PAGE and CXCR5 expression was examined by immunoblot. (**C**) Identification of CXCR4 and CXCR5 coupled to G_α13_ following CXCL13 stimulation. Cell lines were treated with or without CXCL13 and lysed. Antibody against G_α13_ was used to immunoprecipitate (IP) it from total cell lysates. The IP cell lysates were resolved by SDS PAGE and immunoblotted for CXCR5 followed by CXCR4, after stripping. In all the experiments, GAPDH served as loading control. All experiments were repeated three times with unvarying results.

To further validate differences observed in G_α_ subunit(s) coupling and uncoupling to CXCR5 in CXCL13-treated *versus* untreated cells, we separately immunoprecipitated G_αq/11_ and G_αi2_ subunits in untreated and CXCL13-treated PCa cells and immunoblotted for CXCR5. Our results provide the first evidence of multifunctional coupling of CXCR5 to different types of G proteins favoring a pertussis toxin-insensitive signaling pathway mediated by G_αq/11_ in LNCaP cells and a pertussis toxin-sensitive signaling pathway mediated by G_αi2_ in C4-2B and PC3 cells (Figure 3).

### Association of G_α13_ protein, CXCR4, and PAR-1 with CXCR5 in CXCL13-treated PCa cell lines

**Figure 4:**
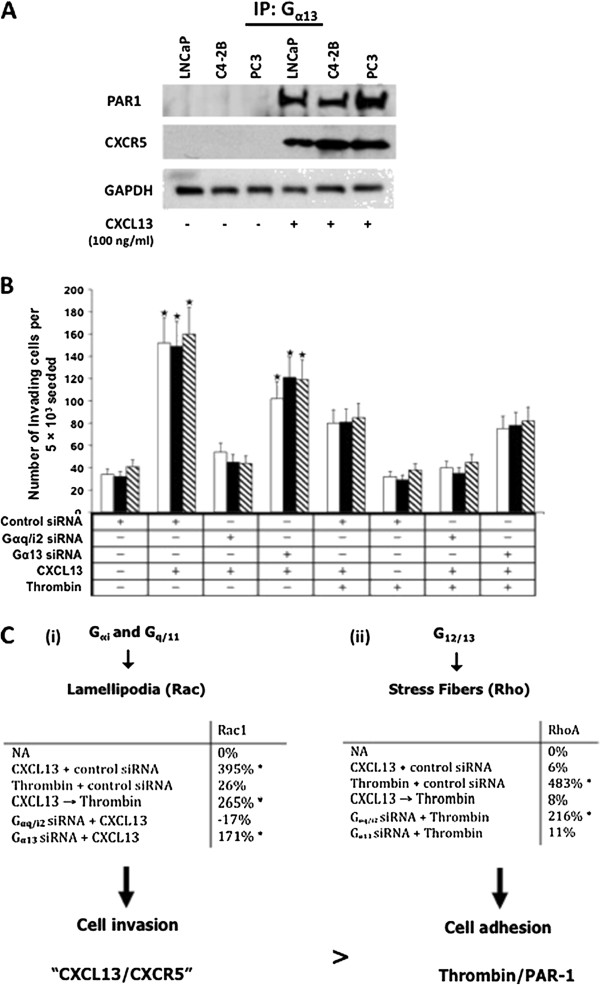
**G**_**α13 **_**association with PAR**-**1 and CXCR5, and G**_**α13 **_**and G**_**αi2 **_**contribution to PCa cell lines invasion and Rac**/**Rho activation.** (**A**) Cell lines were treated with or without CXCL13 and lysed. Antibody against G_α13_ was used to immunoprecipitate (IP) it from total cell lysates. The IP cell lysates were resolved by SDS PAGE and immunoblotted for PAR-1 followed by CXCR5, after stripping. GAPDH served as a loading control. (**B**) Invasion of LNCaP, C4-2B and PC3 cells was assessed using BD Matrigel™ invasion chamber. The assay was performed using LNCaP (open bars), C4-2B (solid bars) and PC3 (hashed bars) cells transfected and/or treated with control siRNA, G_αq/αi2_ siRNA, or G_α13_ siRNA duplex, and CXCL13 and/or Thrombin for 8 h and the cells that migrated to the lower surface of the membrane were counted by microscopy at 40X magnification. CXCL13-treated cells exhibited an enhanced ability to invade Matrigel. Abrogation of G_αq/i2_ decreased the ability of cells to invade whereas silencing of G_α13_ did not affect cell invasion. (**C**) Rac and RhoA protein expression were determined in CXCL13 and/or thrombin treated LNCaP, C4-2B, and PC3 cells. (**i**) shows differential expression of Rac protein, involved in lamellipodia formation, in response to CXCL13, thrombin, CXCL13 followed by thrombin and from cells transfected with G_αq/i2/13_ siRNA in different experiments. (**ii**) shows differential expression of RhoA protein, involved in stress fiber formation and cell adhesion, in response to CXCL13, thrombin, CXCL13 followed by thrombin and from cells transfected with G_α13_ siRNA in different experiments. All experiments were repeated at least three times and results were in accordance with each other.

One surprising result was the association of the G_α13_ subunit with CXCR5 in PCa cell lines treated with CXCL13, but not in untreated cells. Thus, it was critical to confirm this finding by immunoprecipitating G_α13_ protein from CXCL13-treated and untreated PCa cells, and immunoblotting for CXCR5. Results confirm that coupling of G_α13_ to CXCR5 is specific to CXCL13-treated cells (Figure 3C). It has been reported that proteinase activated receptor-1 (PAR-1) is capable of bypassing signaling through G_αi_-pathway to support G_α12/13_-dependent mechanisms, enhancing cellular proliferation, invasion, and metastasis [18]. We therefore examined the association of PAR-1 with G_α13_ and showed that CXCR5 and PAR-1 are linked to G_α13_ following treatment with CXCL13 (Figure 4A).

The presence of CXCR4 in CXCR5 immunoprecipitants (with or without CXCL13 treatment) offers the first evidence of CXCR5 association with CXCR4 (Figure 2B). These interactions could potentially support CXCR4-CXCR5 signaling crosstalk. Moreover, the ability of CXCR4 to engage in G_α13_-mediated cell signaling events that activate Rho pathways leading to cell adhesion has been previously demonstrated [19]. G_α13_ association with CXCR5, CXCR4 and PAR-1 after CXCL13 treatment (Figures 3C &4A) alludes to chemokine receptor oligomer formation or the recruitment of other GPCR-G_α13_ associated signaling complexes after stimulation, which could presumably potentiate synergistic or additional biological events, respectively [20,21].

It is plausible that the CXCL13:CXCR5 axis regulates cell migration by desensitizing CXCR4 and conditional coupling of CXCR5 with PAR-1. Therefore, constitutive coupling of CXCR5 with CXCR4 and PAR-1 after CXCL13 ligation in PCa cells could be another mechanism through which CXCL13 sequesters factors hampering cell migration. To investigate whether this hypothesis holds true, we allowed LNCaP, C4-2B, and PC3 cells previously transfected with G_αq/i2_ or G_α13_ siRNA duplexes to invade across a Matrigel membrane following treatment with CXCL13 or thrombin, which are activating ligands of CXCR5 and PAR-1, respectively. Control siRNA duplex-treated PCa cells exhibited increased invasive potential to CXCL13 (Figure 4B). While abrogation of G_αq/i2_ significantly decreased the ability of cells to invade, silencing G_α13_ did not affect CXCL13-dependent cell invasion. In contrast, PCa cell lines did not invade in response to thrombin alone, but were moderately invasive in the presence of CXCL13 and thrombin. This invasive potential was also G_αq/i2_ -dependent, but G_α13_ -independent. Taken together, these observations suggest CXCL13 is signaling independently of the PAR-1/G_α13_ complex and mainly through CXCR5/G_αq/i2_ to promote PCa cell invasion.

### CXCL13, Thrombin, G_αq/i2_ protein, and G_α13_ protein mediated Rac and RhoA activation in PCa cell lines

G proteins have been shown to differentially activate three members of the Rho family of GTPases (Rac, Cdc42, and RhoA). Our data show that G_αq/11/β3/γ9_ and G_αi2/β3/γ9_ proteins dissociated from CXCR5 after CXCL13 stimulation. This uncoupling is thought to be the result of G protein subunit activation, which stimulates downstream effector molecules, including RhoA and Rac. We therefore performed Rac and RhoA activity assays on CXCL13 and thrombin-treated PCa cells. CXCL13 treatment resulted in a 395% increase in Rac activity, but no change in RhoA activity (Figure 4C). Correspondingly, thrombin-treated PCa cells displayed no significant increase in Rac activity. CXCL13-mediated Rac activation was G_αq/i2_ -dependent, while thrombin-induced RhoA activation was G_α13_ -dependent and G_αq/i2_ -independent. Interestingly, treatment of cells with CXCL13, 5 min before thrombin stimulation did not significantly effect Rac activation, but abrogated thrombin-dependent RhoA activation. Together, our results show CXCL13 stimulation biases PCa cells to invade or migrate, instead of adhere, even in the presence of a potent adherence signal, i.e., thrombin-PAR-1 interactions.

## Discussion

GPCR mediated heterotrimeric G protein signaling is known to regulate cellular motility, growth and differentiation, and gene transcription, three factors central to the biology of cancer. Depending on the physiologic function, expression of G protein(s) subunit isoforms may vary from one cell type to other. G_αi_ subunit inhibits the production of cAMP from ATP. In our study, we found constitutive expression of G_αi_ subunit isoforms in all the cell lines tested. This is in tune with the earlier reports stating that G_αi_ subunit isoforms are the most ubiquitously expressed G protein α isoforms [7,10]. Moreover, studies of tissue samples obtained from patients with T2 stage PCa revealed low levels of G_αs_ subunit compared to high levels in normal controls [22]. G_α12_ and G_α13_ levels were significantly elevated by PC3 and DU-145 cell lines, than compared to PrEC and LNCaP cell lines [23,24]. We found similar results, where G_α12_ was detected only in hormone refractory C4-2B and PC3 cell lines, whereas G_α13_ was significantly elevated in these cell lines. G_β1-4_ and G_γ5,7,9,10_ were expressed in all the cell lines tested. If all of these G_β1-4_ and G_γ5,7,9,10_ proteins could combine to form a dimer, there would be 16 potential arrangements in PCa cells. Emerging evidences suggest that most pairs can indeed form, with some noted exceptions in specific expression systems [4,9,25]. For instance, G_β1_ can combine with G_γ2_ and G_γ5_ but not G_γ3_; and G_β2_ can form a pair with G_γ5_ but not with G_γ1_[26]. Also, G_β3_ pairing with G_γ1_ and G_γ2_ is structurally impossible [9]. G_γ13_ can form stable dimers with G_β1_, G_β3_, and G_β4_, while G_γ10_ is capable of interacting with G_β1_, G_β2_, but not G_β3_[9,27,28]. Future X-ray crystallography studies will be necessary to unravel the precise structural and functional relationship(s) among G protein subunit isoforms.

Malignant cells, which express a wide repertoire of chemokine receptors, respond to chemokines with increased directional migration, proliferation, and/or survival [29]. We have recently demonstrated CXCR5 expression in tissues obtained from PCa patients, and showed that elevated levels of CXCR5 correlate with advanced disease [15]. Furthermore, we established a role for CXCL13 and CXCR5 interaction in prostate tumor progression and elucidated some of the molecular and cellular processes mediated by activation of this chemokine receptor [14]. In confirmation we investigated the expression of CXCR5 and its association with G protein subunits in both androgen sensitive and hormone refractory PCa cells. However, five minutes after CXCL13 stimulation, the G protein subunits (G_αi2_ and G_αq/11_) that bind to CXCR5 were not detected in cell lysates. The plausible explanation for this finding is that binding of CXCL13 to CXCR5 causes conformational changes that elicit the classical dissociation of these G proteins, allowing them to stimulate downstream signaling cascades. Indeed, static and dynamic light scattering measurements of protein complexes will be used to quantify the strength of these interactions, including potential homo- and hetero-associations. In addition to the stoichiometry of these protein-protein associations, future studies will also include isothermal titration calorimetry characterization of these interactions to provide information on the enthalpy, entropy and binding kinetics between these proteins.

Oncogenic mutations of G_αi2_ protein have been identified in ovarian and adrenocortical tumors suggesting a potential role in cellular transformation [30]. G_αi2_ has also been reported to promote B lymphocyte trafficking and motility within lymph nodes in response to CXCL13 [31]. The characteristic G_αi2_ coupling to CXCR5, a chemokine receptor aberrantly expressed by C4-2B and PC3 cell lines, offers a new perspective on the role of G proteins in CXCL13:CXCR5-mediated PCa cell migration.

While the LNCaP cell line is androgen-responsive, C4-2B and PC3 cell lines have hormone-refractory properties [20,32]. This might explain the differential expression of G proteins we observed in LNCaP and C4-2B cell lines, even though the C4-2B cell line was derived from LNCaP cells. Androgen is known to regulate the cellular composition of the normal prostate and acts on a set of specific genes, which impact the protein repertoire of a cell [33]. This dissimilarity in PCa cell line sensitivity to androgen might account for the variation in G protein expression, and could ultimately mandate CXCR5-mediated G protein coupling in these cell types. Our results also suggest that androgen receptor (AR) activation and/or inhibition may contribute to G protein expression in PCa tumors. However, defining the contributions of AR in CXCR5 signaling will be the subject of a different study.

It has been demonstrated that G protein α subunits undergo post-translational lipidation, which increase their affinities for G protein β and γ subunits. These covalent modifications largely determine which G protein α isoforms associate with specific G protein βγ-complexes [34]. Inhibition of the G_βγ_ subunits in general prevents PCa formation and growth *in vivo*[35]. It is worth noting that a polymorphism in the gene encoding G_β3_ subunit is associated with oncogenesis and risk of bone metastasis in patients with breast cancer, while the homozygous G_β3_ genotype conferred protection against disease progression [36]. Hence, the identification of G_β3/γ9_ coupling to CXCR5 is of considerable interest and the functional relevance of this finding is a matter for future studies. It has also been noted that free G_βγ_ complexes can effect other second messengers, e.g., phospholipase A2 and phospholipase C, or gating ion channels, e.g., G protein coupled inward rectifying potassium channels and L-type calcium channels. While this has not been observed following CXCR5 signaling, future studies will be needed to determine the potential signaling events induced by the G_β3-γ9_ complex following CXCR5 stimulation.

We also found that G_α13_ protein associates with CXCR5 following CXCL13 stimulation. While multiple scenarios could exist to explain this result, G_α13_ association with active CXCR5 could be the product of ligand-mediated G protein switching. It has been reported that G protein isoforms switch their coupling to receptors in response to ligand binding in a cAMP-dependent protein kinase (PKA) fashion to presumably initiate a new set of signaling cascades [37]. This phenomenon has been described in CHO cells, where the β_2_-adrenergic receptor switches its coupling specificity from G_αs_ to G_αi_ in response to agonist binding [38].

**Figure 5:**
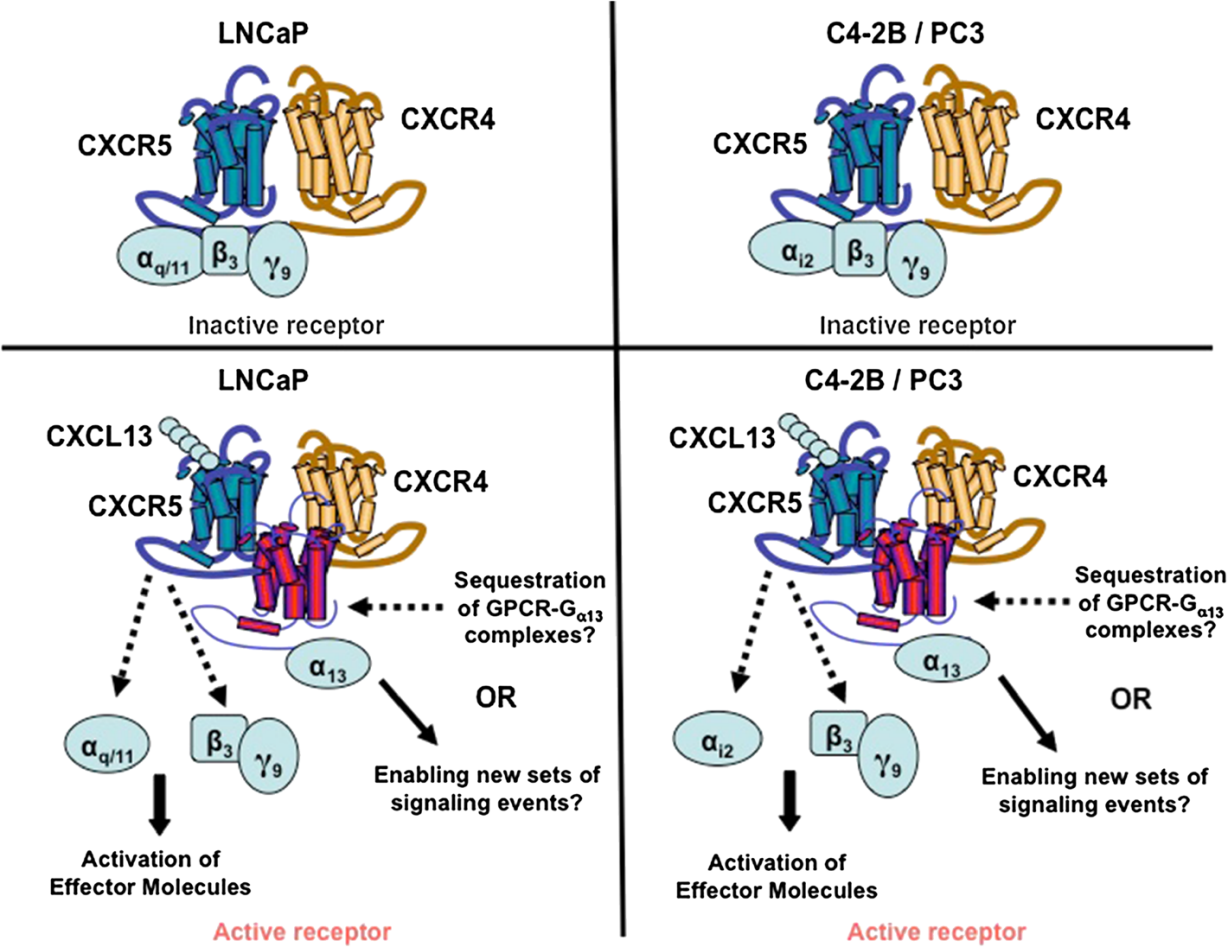
**Hypothetical model of CXCR5 interactions in PCa cells.** CXCR5 associates with CXCR4 and couples with G_αq/11/_G_β3/_G_γ9_ heterotrimers in androgen-dependent LNCaP cell line or G_αi2/_G_β3/_G_γ9_ heterotrimers in hormone refractory C4-2B and PC3 cell lines in the absence of its specific ligand, CXCL13. Upon CXCL13 stimulation, G proteins dissociate from CXCR5 to activate effector molecules. In addition, CXCL13-activated CXCR5 associates or sequesters G_α13_protein favoring signals that would promote PCa cell motility.

Previously it has been shown that CXCR4 is widely expressed by PCa cell lines and migration and invasive potential of these cells were significantly impaired by anti-CXCR4 antibodies [39]. In our study, we found a constitutive coupling of CXCR4 to CXCR5 and a likely oligomerization with other GPCRs upon CXCR5 activation (Figure 5). This interaction can sequester G_α13_ and/or associated receptors to apparently diminish their functions, e.g. adhesion. While co-immunoprecipitation is considered the gold standard for determining protein-protein interactions of endogenous untagged proteins, futures studies will be needed to ascertain the affinity and confirmation of these interactions. Indeed, it will be important for potential molecular drug development efforts to determine the binding constants and the precise regions where CXCR5 (or CXCR4) and G_αq/11_, G_αi2_, G_α13_, G_β3_ and G_γ9_ proteins interact.

The ability of GPCRs to differentially couple to multiple classes of G proteins (G_αi_, G_αq/11_, G_α12/13_) has also been described for sphingosine-1-phosphate receptors, and the liver pancreastatin receptor [40,41]. While the possibility of CXCR5 switching from G_αi_ to G_α13_ signaling pathways requires further investigation, the possibility of its occurrence presents a means for tumor cells to acquire new signaling machinery that could promote disease progression. Hence, it is more likely that CXCR5 binds G_α13_ protein as a mechanism to sequester and prevent it from signaling, which would favor Rac > > RhoA activation and cell migration. To explain, G_α12/13_ family of G proteins have been shown to stimulate RhoA activation and subsequent actin cytoskeletal rearrangements characterized by the formation of stress fibers for focal adhesion [42].

RhoA activation causes the formation of stress fibers and focal adhesions. Rac activation leads to lamellipodia formation and membrane ruffling, while cdc42 activation results in filopodia formation. These cellular processes are particularly important for cell migration and adhesion [43]. Compelling evidence suggest that Rac are primarily activated by G_αi_ and G_αq_ subunits [44]. RhoA has shown to be activated downstream of G_α12/13_ subunits and to a lesser extent by G_αq_, while G_βγ_ complexes are thought to contribute to activation of both RhoA and Rac pathways through direct stimulation of PI3K [45].

## Conclusions

We show differential G protein expression by PCa cell lines and establish specific heterotrimeric coupling to CXCR5 in an androgen-sensitive (LNCaP) and hormone refractory (C4-2B and PC3) manner. We also provide evidence for G_α13_ protein association with CXCR5 following CXCL13 stimulation, which could inhibit or potentiate various cellular processes. Moreover, we identify for the first time the constitutive coupling of CXCR4 to CXCR5. Clearly, there is much to learn about how specific heterotrimeric G protein compositions are regulated, and how these associations dictate unique signaling pathways. It will also be important to determine the clinical relevance of the G_αq/11/_G_β3/_G_γ9_ heterotrimer in early and G_αi2/_G_β3/_G_γ9_ in advanced or hormone refractory PCa.

Several observations have described chemokine receptor oligomer formation resulting in unusual G protein signaling [46]. The hetero-dimerization between CCR2 and CCR5 has been extensively explored and suggests a mechanism of differential receptor coupling to pertussis toxin-sensitive to -insensitive G proteins [47,48]. Evidence also supports the ability of CCR5 to interact with non-chemokine receptors including opioid receptors [49]. While CXCR4 is present in almost all invasive cancers, CXCR5 has been implicated in advanced stages of chronic myelogenous leukemia, head and neck cancers, colon, and prostate cancer [1,12,29,50]. There is growing evidence to suggest transactivation of chemokine receptors will result in signal amplification at the receptor level, providing a means for tumor cells to metastasize and grow [21,46].

The signaling cascade following CXCL13-CXCR5 interactions is indeed complex. These signals support Rac activation and invasion in a G_αq/i2_ protein dependent fashion. Further, CXCR5 associates with CXCR4 and following activation can sequester G_α13_ and/or associated receptors to seemingly diminish their functions.

No doubt, CXCR5 and/or CXCL13 blockade and specific G protein inhibition might prove to be effective therapeutic strategies to disrupt CXCR5 (and possibly CXCR4) signaling to abrogate PCa cell metastasis.

## Methods

### Cell lines and culture

Human prostate cancer cell lines (LNCaP, C4-2B, and PC3) and the epithelial cell line RWPE-1 derived from normal prostate were used in this study. All the cell lines were obtained from ATCC. To authenticate the cell lines, we carried out short tandem repeats genotyping. RWPE-1 cell line (ATCC # CRL-11609) is an established normal prostate epithelial cell line that was cultured in keratinocyte serum free media (K-SFM) supplemented with bovine pituitary extract (0.05 mg/ml) and epidermal growth factor (5 ng/ml) at 37°C in a humidified atmosphere with 5% CO_2._ LNCaP cell line (ATCC # CRL-1740) is derived from the left supraclavicular lymph node of a metastatic prostate adenocarcinoma patient and is responsive to 5-alpha-dihydrotestosterone. C4-2B cell line is derived from the LNCaP cell line; however, it is hormone refractory. The PC3 cell line (ATCC # CRL-1435) was derived from a bone metastasis of a grade IV prostatic adenocarcinoma patient. All three PCa cell lines were cultured in complete RPMI 1640 media supplemented with 10% fetal bovine serum (FBS) and maintained in a cell culture incubator at 37°C in a humidified atmosphere with 5% CO_2_. Cell lines were serum starved overnight prior to treatment with 100 ng/ml of CXCL13 (Pepro Tech, NJ, USA) or 1U/ml of thrombin (Sigma, MO, USA).

### Immunoprecipitation

RWPE-1, LNCaP, C4-2B and PC3 cells were lysed in a cell lysis buffer containing 1% NP40, 1% Triton X-100, 0.25% deoxycholate, 100 mM NaCl, 50 mM Tris–HCl, pH7.4, and protease and phosphatase inhibitors (Roche, IN, USA). The protein concentrations of whole cell lysates were determined by bicinchoninic acid (BCA) protein determination assay (Pierce, IL, USA). To determine selective G protein isoforms coupled to CXCR5, equal amounts (100 μg) of LNCaP, C4-2B, and PC3 cell lysates were incubated with 1 μg of mouse anti-CXCR5 (R&D systems, MN, USA), mouse anti-G_αi2_, rabbit anti-G_αq/11_, or goat anti-G_α13_ antibodies (Santa Cruz, CA, USA) for 2 h at 4°C. Immune complexes were collected by adding 20 μl of Agarose A/G PLUS beads (Santa Cruz, CA, USA) overnight at 4°C. Following incubation protein complexes were washed twice with lysis buffer by centrifugation at 10,000 × *g* for 10 min at 4°C and released from the beads by boiling in sample buffer for 5 min. The resultant immunoprecipitates were further analyzed by immunoblot analysis.

### Immunoblotting and antibodies

Western blot analysis was conducted on immuno − precipitants generated as described above or directly on cell lysates containing 50 μg of protein. Samples were denatured by boiling in Laemmli buffer for 5 min, resolved by electrophoresis on 4-15% gradient SDS-polyacrylamide gel as needed, and transferred to nitrocellulose membranes using a semi-dry transfer cell system (Bio-Rad, CA, USA). Membranes were blocked for 1 h at room temperature (RT) in 5% non-fat milk in 1X TTBS (30 mM Tris-Base, 150 mM NaCl, and 0.1% Tween 20), followed by washing with 1X TTBS. Primary antibodies against G proteins α_i1_, α_i2_, α_i3_, α_s_, α_q/11_, α_12_, α_13_, α_16_, β_1_, β_2_, β_3_, β_4_, β_5_, γ_1_, γ_2_, γ_3_, γ_4_, γ_5_, γ_7_, γ_9_, γ_10_, γ_13_, CXCR5 (Santa Cruz, CA, USA), and CXCR4 (R&D systems, MN, USA) were added to the membranes and incubated overnight at 4°C in 5% non-fat milk. Membranes were then washed and corresponding horseradish peroxidase (HRP)-conjugated secondary antibodies (Santa Cruz, CA, USA) were added for 1 h followed by additional washes. Immunoreactive proteins were visualized by a chemiluminescent detection reagent (Amersham, PA, USA) on autoradiographic films. The blots were re-probed each time to stain different G protein subunit isoforms. Following development for G proteins, all membranes were stripped and re-probed with antibody against GAPDH (Ambion, NY, USA) to ensure equal loading.

### Invasion assay

PCa cell invasion was assessed using BD Matrigel™ invasion chamber (BD Biosciences). Briefly, Matrigel inserts were hydrated for 2 h with 500 μl of DMEM at 37°C with 5% CO_2_. CXCL13 (100 ng/ml) or thrombin (1 U/ml) was added to the bottom chamber containing serum-free RPMI medium. LNCaP, C4-2B, and PC3 cells were transfected with 1 μg control siRNA, G_αq/i2_ siRNA, or G_α13_ siRNA duplex (Santa Cruz, CA, USA) prior to harvest, and added to the top chambers in serum-free RPMI medium at 10,000 cells per well. The cells were allowed to invade for 8 h at 37°C with 5% CO_2_. Non-invading cells on the upper surface of the membrane were removed with a cotton swab. The cells that migrated to the lower surface of the membrane were fixed with methanol at RT for 5 min, stained with crystal violet for 2 min, and washed with distilled water. The membranes were peeled and mounted on glass slides. Cells were then counted by microscopy at 40X magnification. Experiments were performed in triplicate and repeated three times.

### Rac and RhoA G-LISA activation assays

Rac and RhoA activity were determined from cell lysates collected from LNCaP, C4-2B, and PC3 cells treated with or without CXCL13, thrombin, control siRNA, G_αq/i2_ siRNA and/or G_α13_ siRNA. PCa cells were transfected with 1 μg of control, G_αq/i2_ siRNA, or G_α13_ siRNA duplexes (Santa Cruz, CA, USA) as before. Optimal knockdown of RNA and resulting protein knockdown occurred 72 h after transfection, which was confirmed by RT-PCR and Western blot analysis. Transfected PC3 cell cultures were pre-treated with media alone, 100 ng/ml of CXCL13 or 1 U/ml of thrombin for 30 min. Subsequently, cultures were treated with these CXCR5 or PAR-1 ligands to determine Rac and RhoA activities. After 10 min. of stimulation, protein lysates were isolated and assayed using the colorimetric-based G-LISA™ Rac activity and luminescence-based G-LISA™ RhoA activation assay kits (Cytoskeleton, CO, USA), according to the manufacturer’s instructions. Briefly, proteins were isolated using the provided cell lysis buffer and lysates were collected by centrifugation at 10,000 rpm at 4°C for 2 min. Protein concentrations from each sample were quantified and then adjusted to contain protein concentrations of 2 mg/ml for the assay. Absorbance and luminescence were detected as suggested by the manufacturer. Changes in Rac and RhoA activity among conditions are reported as fold difference normalized to the sample with no additions.

## Abbreviations

ATCC: American Type Culture Collection; BCA: Bicinchoninic acid; GPCRs: G protein coupled receptors; FBS: Fetal bovine serum; HRP: Horseradish peroxidase; PCa: Prostate cancer; PI3K: Phosphoinositide-3 kinase; RPMI: Roswell Park Memorial Institute; SDS-PAGE: Sodium dodecyl sulfate-polyacrylamide gel electrophoresis; TTBS: Tris-Tween Buffered Saline.

## Competing interests

The authors declare that they have no competing interests.

## Authors’ contributions

CH carried-out all experiments, quantified protein levels, and analyzed data with the assistance of PS, RS, PG, DT, and SS. JL conceived the study, participated in its design with all authors, coordinated and helped to draft the manuscript with the assistance of all authors. All authors read and approved the final manuscript.
